# Dysregulation of the JNK signaling pathway in tuberculosis: mechanisms and therapeutic strategies

**DOI:** 10.3389/fcimb.2025.1663992

**Published:** 2025-11-26

**Authors:** Yushan Yao, Kang Li, Yinghui Chai, Xianping Deng, Min Li, Junya Lan, Yan Liang, Xueqiong Wu, Hong Lei

**Affiliations:** 1 The Eighth Medical Center of PLA General Hospital, Beijing, China; 2 Hebei North University, Zhangjiakou, China

**Keywords:** MTB, JNK signaling pathway, immune response, immune evasion, inflammation, therapeutic strategies, JNK inhibitors

## Abstract

Tuberculosis (TB), which is caused by *Mycobacterium tuberculosis (*Mtb), remains a major infectious disease worldwide. Despite the availability of anti-TB drugs, the emergence of drug resistance, the need for prolonged treatment duration and the occurrence of side effects highlight the urgent need for new therapeutic strategies. The c-Jun N-terminal kinase (JNK) signaling pathway, which is an important member of the mitogen-activated protein kinase (MAPK) family, plays a crucial role in regulating cellular stress responses, inflammation, apoptosis, autophagy, and ferroptosis. Excessive JNK activation can induce uncontrolled inflammation, tissue damage, and chronic immune activation. In contrast, insufficient activation may impair the host’s defense, facilitating Mtb immune evasion and persistence. Such alterations disrupt the delicate immune equilibrium essential for effective pathogen clearance and host protection. This review summarizes the molecular mechanisms through which Mtb manipulates the JNK signaling pathway to disrupt host immunity, emphasizing its roles in metabolic reprogramming, apoptosis, autophagy, and ferroptosis. In addition, this review discusses potential therapeutic strategies targeting the JNK pathway, including the development of selective JNK inhibitors, with a focus on their prospects in TB treatment. Progress has been made in elucidating the role of JNK signaling pathway in TB, but further research is required to clarify its specific mechanisms and evaluate the safety and efficacy of JNK-targeted interventions. Continued exploration of this pathway may provide new targets and strategies for TB therapy.

## 1 Introduction

Tuberculosis(TB), which is caused by *Mycobacterium tuberculosis*(Mtb), remains a major global public health threat. According to the 2024 World Health Organization (WHO) Global Tuberculosis Report, an estimated 10.8 million people developed TB worldwide in 2023, with 1.25 million deaths attributed to the disease (World Health, 2024). The highest TB burdens are found in South-East Asia (46%) and Africa (32%) regions, together accounting for over 95% of global cases, followed by the Western Pacific (18%). While the global incidence of TB declined by 8.3% between 2015 and 2023, this reduction falls far short of the WHO’s End TB Strategy target of a 50% reduction by 2025. The report also emphasizes that Africa continues to face substantial challenges in TB prevention and control, with persistent gaps in diagnostic capacity and treatment coverage (World Health, 2024). Despite notable advances in TB prevention and management in recent years, multiple obstacles still hinder the effective control of the disease. The emergence and spread of drug-resistant TB, including multidrug-resistant TB (MDR-TB), and extensively drug-resistant TB (XDR-TB), prolonged treatment duration, severe drug-related adverse effects, and poor patient compliance continue to undermine treatment success (Singh and Chibale, 2021). These persistent challenges highlight the urgent need to develop new therapeutic strategies and molecular targets to enhance TB treatment outcomes.

**Figure 1:**
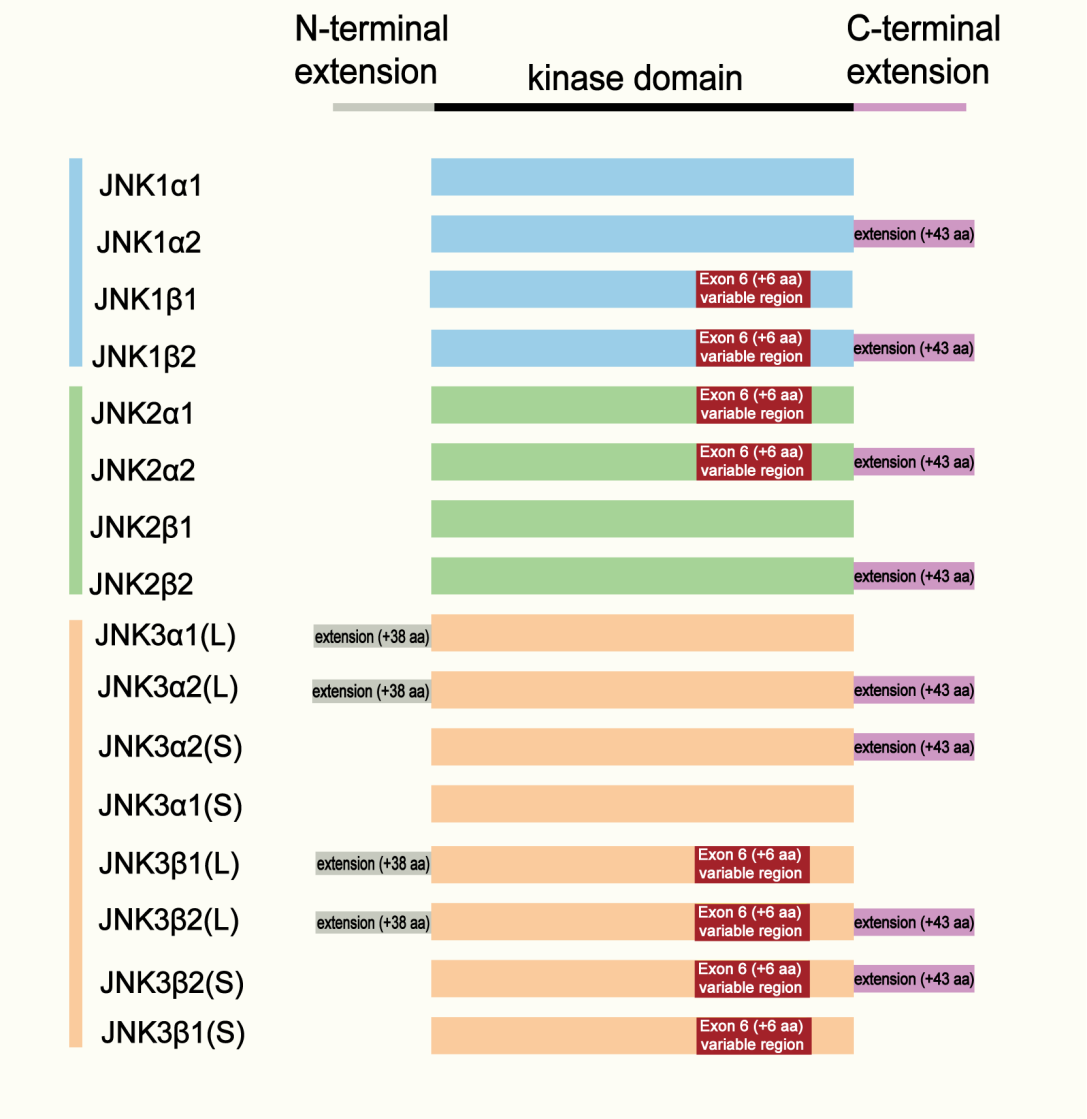
Structural organization and isoform diversity of JNK family members.

The c-Jun N-terminal kinase (JNK), also known as the stress-activated protein kinase (SAPK), is an important member of the mitogen-activated protein kinase (MAPK) family. The JNK subfamily comprises three genes, *MAPK8, MAPK9 and MAPK10*, which encode JNK1, JNK2 and JNK3 isoforms, respectively. A schematic representation of the three isoforms is shown in **Figure 1**. All JNK proteins contain a characteristic serine/threonine (Ser/Thr) protein kinase domains (Bogoyevitch and Kobe, 2006; Kumar et al., 2015). JNK1 and JNK2 are widely expressed in tissues and cells including immune cells such as macrophages. In contrast, JNK3 is predominantly expressed in the brain, heart and testes (Hotamisligil, 2006; Bode and Dong, 2007). As an important branch of the MAPK signaling pathway, the JNK pathway plays a key role in multiple physiological and pathological processes, including cell proliferation, differentiation, apoptosis, stress responses and immune regulation.

Accumulating evidence indicates that the JNK signaling pathway is critically involved in the pathogenesis of diverse infectious diseases, including viral, bacterial, and fungal infections. Its role in respiratory infections has received particular attention in recent years (Bennett, 2006). Emerging studies have revealed that JNK also contributes to the initiation and progression of TB (Paik et al., 2019). For example, the Mtb-secreted effector EST12 activates the JNK-AP-1-Myc pathway to induce the production of pro-inflammatory cytokines and nitric oxide, thereby enhancing Mtb clearance (Wu et al., 2022). Similarly, PE17 modulates inflammatory cytokine levels through JNK signaling, leading to reduced Mtb survival within macrophages (Abo-Kadoum et al., 2021a). In addition, baicalin, a bioactive flavonoid, alleviates inflammatory injury by inhibiting the excessive activation of the ERK/JNK axis (Duan et al., 2021). Taken together, these findings suggest that appropriate activation of JNK signaling by Mtb contributes to host defense and facilitates pathogen clearance.

This review provides a narrative overview of the impact of Mtb infection on the JNK signaling pathway. It focuses particularly on how the dysregulation of this pathway contributes to tuberculosis pathogenesis and its potential as a therapeutic target. Additionally, we summarize the significant knowledge gaps in existing research and future directions, to offer new perspectives and strategies for developing innovative TB therapies.

Schematic representation of the structural domains and alternative splicing variants of JNK1, JNK2, and JNK3. Each isoform contains a conserved kinase domain (black bar) flanked by an N-terminal extension and a C-terminal extension. Alternative splicing of exon 6 (resulting in α and β variants) and inclusion of the C-terminal extension (+43 amino acids) generate four major isoforms for each JNK gene (α1, α2, β1, β2). JNK3 isoforms further differ by the presence of a long (L, + 38 aa) or short (S) N-terminal extension. The color coding indicates JNK subtypes: blue (JNK1), green (JNK2), and orange (JNK3).

## 2 Methods

To provide a comprehensive and representative overview of the role of JNK in tuberculosis, we conducted a structured literature search. Although this is a narrative review, we used a systematic approach to identify relevant studies.

### 2.1 Search strategy

We performed electronic searches in the PubMed and Web of Science databases for articles published from inception until July 2025. The search strategy used a combination of the following keywords and Boolean operators: (“JNK” or “c-Jun N-terminal kinase” or “MAPK,” or “autophagy,” or “ferroptosis,” or “host-directed therapy”) and (“tuberculosis” or “TB” or “M. tuberculosis”). Additionally, we manually screened the reference lists of the retrieved articles and relevant reviews to identify any additional pertinent publications.

### 2.2 Study selection and eligibility criteria

The identified records were screened based on their titles and abstracts, followed by a full-text assessment of potentially eligible articles. Studies were included if they were original research articles published in English that directly investigated the function, activation, or regulation of the JNK signaling pathway in the context of M. tuberculosis infection or TB pathogenesis, either *in vitro*, *in vivo*, or in human subjects. Articles that only mentioned JNK incidentally without substantive investigation, or those not directly related to the core topic, were excluded.

## 3 Overview of the JNK signaling pathway

### 3.1 Structure and activation mechanism

The JNK signaling pathway is an important member of the MAPK family. It mainly transmits signals through a series of kinase cascade reactions and plays a key role in processes such as cell stress, inflammation, apoptosis and immune response. Its activation and signal transduction process usually involves the following key steps: (1) Signal stimulation: Activation of the JNK signaling pathway can be triggered by a variety of intracellular stimuli (e.g., oxidative stress, DNA damage, and osmotic stress), intercellular stimuli (e.g., growth factors and cytokines), and extracellular stimuli (e.g., pathogen infection and inflammatory responses). These stimuli initiate signaling through the engagement of various cell surface receptors, such as G protein-coupled receptors (GPCRs), Wnt receptors, transforming growth factor β (TGF-β) receptors, tumor necrosis factor (TNF) receptors and Toll-like receptors (TLRs), further triggering downstream intracellular kinase cascades (Zeke et al., 2016). (2) Activation of upstream kinases and phosphorylation: Activation of JNK depends on the cascade phosphorylation reaction of upstream kinases. First, upstream MAP kinase kinase kinases (MAP3Ks), such as ASK1 and MEKK1, are activated, which then phosphorylate and activate the MAP kinase kinases (MAP2Ks), mainly MKK4 and MKK7 (Zeke et al., 2016). These MAP2Ks then phosphorylate JNK at threonine and tyrosine within the TPY motif (Thr183/Tyr185), leading to a conformational change that converts JNK from an inactive state to an active form (Fleming et al., 2000; Lisnock et al., 2000). This dual phosphorylation event represents the central step in JNK activation. (3) Translocation and function: Activated JNK translocates to the cell nucleus through the mediation of several important proteins importin-β family members (e.g., importin-3, importin-7, and importin-9) or α-importin (Misheva et al., 2014; Zehorai and Seger, 2018). In the cell nucleus, JNK phosphorylates several transcription factors (e.g., c-Jun, ATF2, SMAD, and c-Myc), altering their activity and stability, thereby regulating the expression of downstream genes, and further affecting various biological processes such as cell proliferation, differentiation, apoptosis, inflammatory response, and fibrosis.

### 3.2 Negative regulation of JNK activity

**Figure 2:**
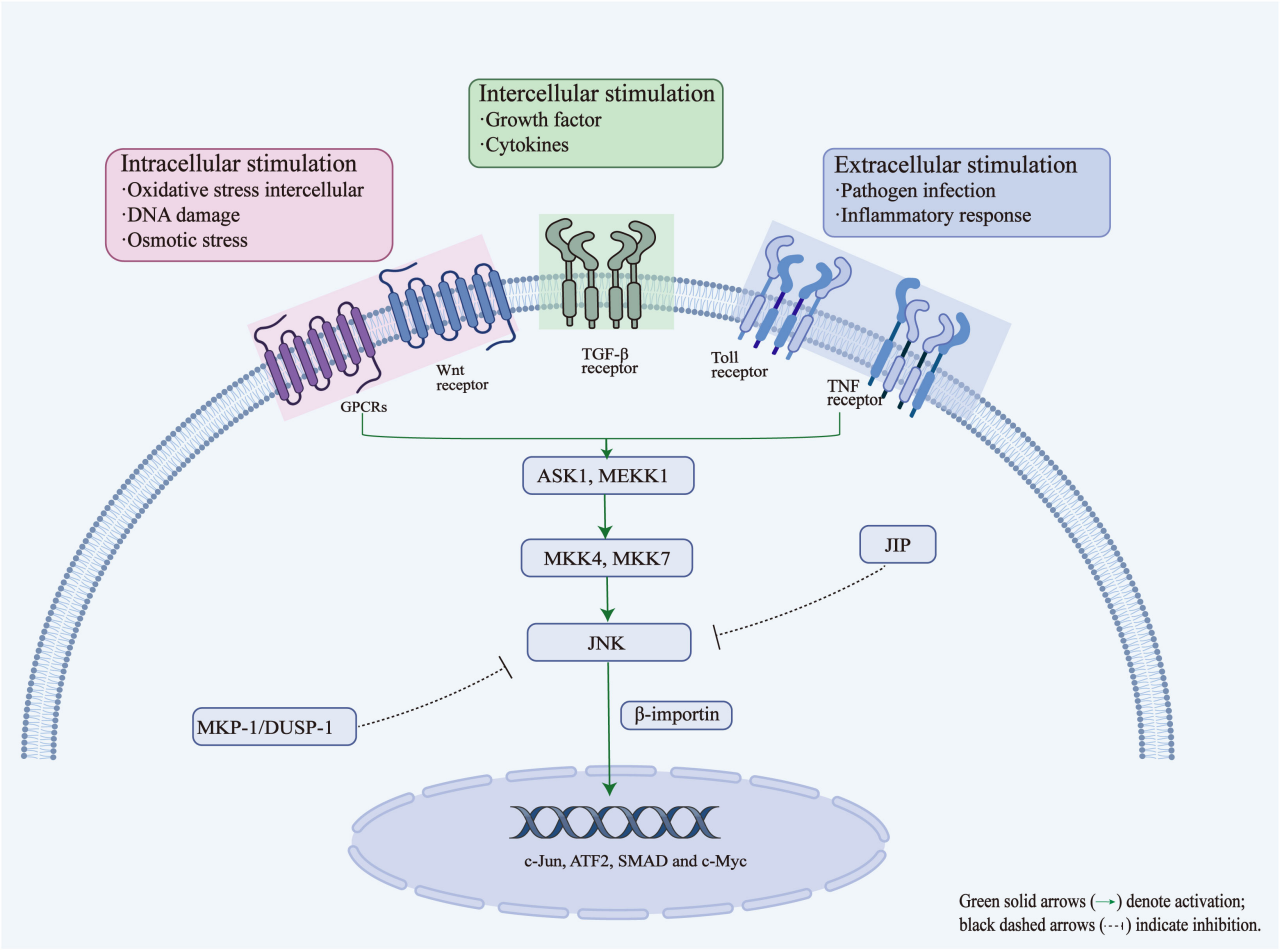
Activation and regulatory mechanisms of the JNK signaling pathway.

The JNK signaling pathway is tightly controlled by multiple negative regulatory mechanisms to prevent excessive activation, which could result in cellular damage and pathological responses. The major mechanisms of JNK inhibition include the following: (1) Regulation by MAPK phosphatases (MKPs): specific MAPK phosphatases, such as MKP-1 (also known as DUSP1), can dephosphorylate and inactivate JNK, thereby attenuating downstream signaling and modulating cellular stress and survival responses (Staples et al., 2010). Notably, activated JNK can induce the expression of its own negative regulators, such as MKP-1, forming an autoregulatory negative feedback loop that helps maintain the balance of intracellular signals (Awano et al., 2008; Li et al., 2010). This feedback mechanism ensures a rapid and transient JNK response to stress stimuli. (2) JNK inhibitory proteins (JIPs): scaffold or inhibitory proteins, such as those in the JIP family, can bind directly to JNK and prevent its activation (Zeke et al., 2016). They inhibit JNK signaling by blocking the interaction between JNK and its upstream kinases (e.g., MKK4/7) or downstream substrates (e.g., c-Jun), thereby restraining excessive signal amplification. Such scaffold-mediated inhibition is essential for the spatial and temporal control of JNK signaling. (3) Other post-translational regulatory mechanisms: In addition to phosphorylation, JNK activity is fine-tuned by other post-translational modifications, such as ubiquitination and acetylation. For example, some E3 ubiquitin ligases can promote the ubiquitination and subsequent proteasomal degradation of JNK, thereby decreasing its cellular abundance and limiting its activity (Zeke et al., 2016). These layers of regulation help to maintain intracellular homeostasis while avoiding prolonged inflammatory or apoptotic responses. Overall, these negative regulatory processes ensure that JNK signaling remains transient and precisely modulated under physiological conditions. A deeper understanding of these mechanisms is important for developing selective JNK inhibitors as potential therapeutic agents (Li et al., 2021). **Figure 2** shows the genetic and molecular mechanisms involved in JNK signal regulation.

The JNK signaling pathway can be activated by various stimuli, including intracellular stress signals (e.g., oxidative stress, DNA damage and osmotic stress), intercellular mediators (e.g., growth factors and cytokines), and extracellular stimuli (e.g., pathogen infection and inflammatory responses), which activate JNK through a cascade involving upstream kinases such as ASK1/MEKK1 and MKK4/MKK7. Once activated, JNK translocates to the nucleus via importin-β, where it phosphorylates transcription factors such as c-Jun, ATF2, SMAD, and c-Myc. This regulates the expression of genes related to stress, apoptosis, and inflammation. JNK is negatively regulated through MAPK phosphatases (MKP-1/DUSP1), which dephosphorylate JNK, and inhibitory scaffold proteins such as JIP, which block the interaction between JNK and its upstream or downstream partners. These feedback and inhibitory mechanisms ensure signaling homeostasis and prevent excessive cellular responses.

### 3.3 Regulatory role of the JNK signaling pathway in the immune response

**Figure 3:**
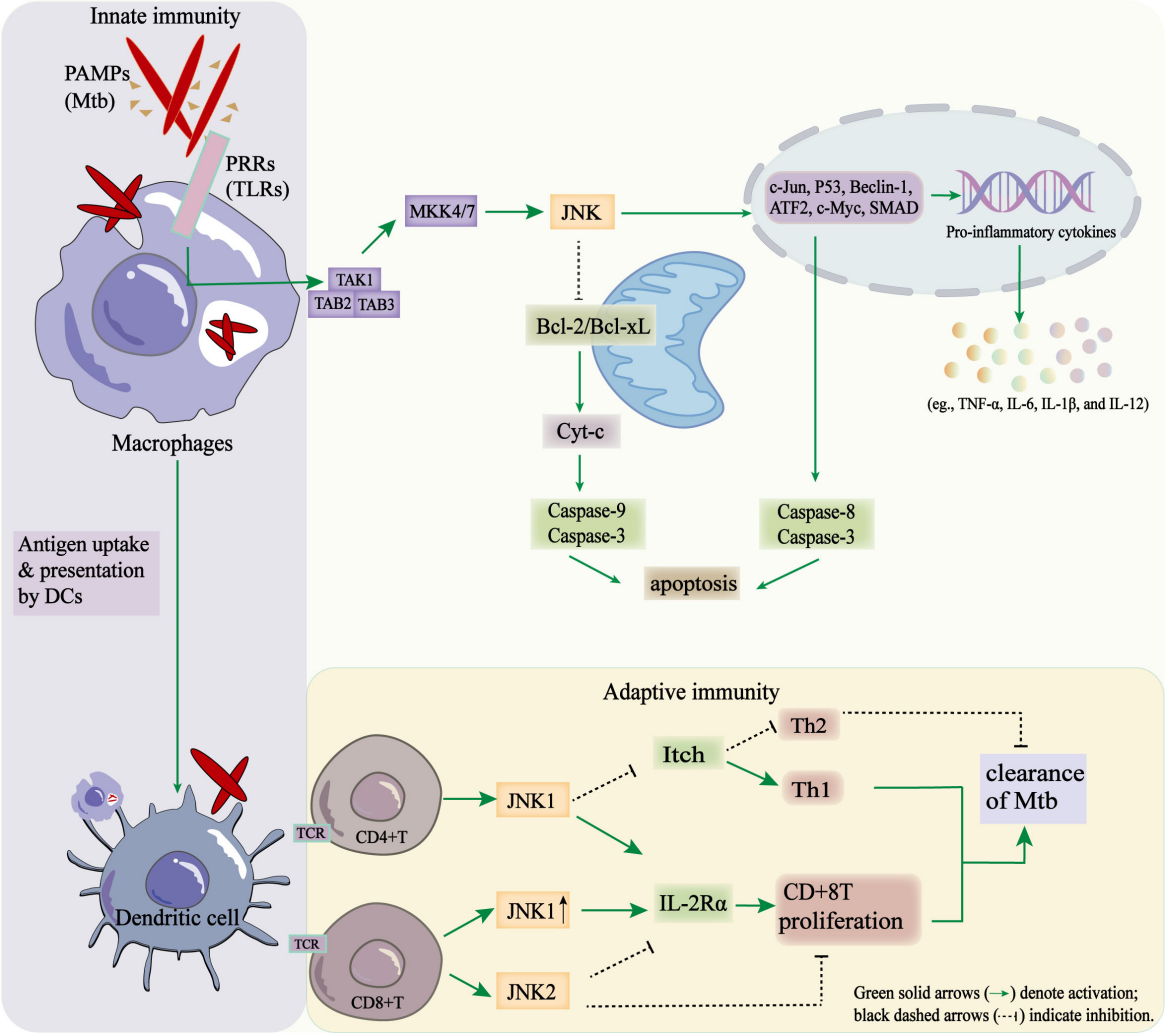
Regulatory role of the JNK signaling pathway in coordinating innate and adaptive immune responses during Mtb infection.

The JNK signaling pathway plays an important regulatory role in both innate and adaptive immune responses. It plays a role in both the initial defense of the host against pathogens and the establishment of immune memory, the activation, differentiation and effector function of immune cells (**Figure 3**). A comprehensive understanding of the immunoregulatory mechanisms of the JNK pathway are essential for identifying novel immunotherapeutic targets and optimizing host-directed therapies against infectious diseases such as TB.

#### 3.3.1 Role in innate immunity

In the innate immune response, activation of the JNK signaling pathway promotes inflammation, pathogen clearance and the regulation of cell fate by controlling cytokine production, autophagy, and apoptosis in a coordinated manner. Upon infection by bacteria, viruses, or other pathogens, pathogen-associated molecular patterns (PAMPs) recognized by pattern recognition receptors (PRRs), including Toll-like receptors (TLRs) and NOD-like receptors (NLRs), which are expressed on macrophages and dendritic cells. This engagement triggers MAPK cascades that culminate in JNK activation. Activated JNK then phosphorylates transcription factors such as c-Jun and ATF2, thereby promoting the transcription of pro-inflammatory cytokines, such as tumor necrosis factor (TNF-α), IL-6, IL-1β, and IL-12. These cytokines amplify local inflammatory responses and induce the expression of chemokines. This, in turn, recruits additional immune effector cells to the site of Mtb infection, thereby enhancing the containment of Mtb. Cytokines contribute indirectly to immune cell recruitment through their pro-inflammatory potential (Medzhitov and Janeway, 1997; Dong et al., 2002). In addition, JNK plays an important role in regulating autophagy and apoptosis during infection. Activation of JNK promotes the formation of autophagosomes and the clearance of pathogens by regulating autophagy-related genes, such as Beclin-1 and ATG5. This links stress signaling to the elimination of intracellular Mtb via xenophagy. During severe or persistent infections, JNK signaling can induce apoptosis of infected macrophages, thereby limiting pathogen replication and dissemination (Chen et al., 1996; Tournier et al., 2000; Chen et al., 2021). Mechanistically, JNK mediated apoptosis proceeds via two primary routes: First, nucleus, translocation of JNK leads to the phosphorylation of transcription factors, such as c-Jun and p53. This induces the expression of AP-1 or p53-dependent pro-apoptotic genes, as well as the activation of caspase-8 and caspase-3 (Dhanasekaran and Reddy, 2008; Tang et al., 2012); Secondly, mitochondrial JNK signaling promotes apoptosis by phosphorylating and inactivating the anti-apoptotic proteins Bcl-2 and Bcl-xL. This facilitates Bid-Bax-dependent cytochrome c, and the activation of caspase-9 and caspase-3 (Dhanasekaran and Reddy, 2008).

Further emerging studies indicate that JNK signaling integrates innate immune activation with metabolic reprogramming in macrophages. For instance, JNK activation via the JNK/COX-2/HIF-1α axis increases glycolysis, thereby promoting M1 macrophage polarization and enhancing their microbicidal capacity (Zhang et al., 2023). Broader reviews propose that JNK orchestrates key links between stress signaling and metabolic pathways in immune cells, acting as a signaling-metabolism hub (Yan et al., 2024). Therefore, JNK may act as a dual regulator of innate immunity: while it facilitates pathogen clearance, hyperactivation could exacerbate immunopathology.

#### 3.3.2 Role in adaptive immunity

The JNK signaling pathway interacts with upstream microRNAs to regulate T cell responses and modulates adaptive immune cells through diverse downstream effectors. During Mtb infection, activation of the TLR2–NF-κB–JNK axis induces the expression of miR-155-5p (Ghorpade et al., 2012). Notably, upregulation of miR-155-5p plays a protective effect in TB by prolonging the survival of infected macrophages and dendritic cells, which enhances their activation and promoting T cells recruitment, thereby linking innate and adaptive immunity (Rothchild et al., 2016; Etna et al., 2018).

TAK1, as an upstream kinase of JNK, integrates T cell receptor (TCR) and cytokine signals in both naive and effector T cells to activate JNK and regulate antigen-specific T cell responses (Wan et al., 2006). Upon the co-stimulation of TCR/CD28, MKK4/7 phosphorylation activates JNK, thereby regulating the survival, differentiation, and proliferation of T cells (Su et al., 1994; Dong et al., 2000; Lanna et al., 2017). JNK1 promotes T cell proliferation by upregulating the expression of the IL-2 receptor alpha chain (IL-2Rα), enhancing IL-2 signaling and supporting the clonal expansion of activated T cells (Dong et al., 2000). In addition, the MEKK1–JNK1–Itch signaling axis inhibits the production of Th2-type cytokines (e.g. IL-4, IL-5 and IL-13), and promotes Th1 differentiation, enhancing IFN-γ-mediated macrophage activation and bacterial clearance (Gao et al., 2004; Venuprasad et al., 2006). JNK signaling is also essential for optimally activating and enabling the effector function of CD8^+^ T cells (Wang et al., 2014). JNK1 enhances IL-2Rα expression, promoting cell proliferation; conversely, JNK2 inhibits excessive expansion of CD8^+^ T cell, thereby preventing immunopathology (Conze et al., 2002). Consistently, JNK deficiency results in impaired antiviral CD8^+^ T cell responses and defective viral clearance (Wang et al., 2014).

In B cells, JNK signaling plays a key role in the survival, activation, and differentiation. MEKK1-dependent JNK activation promotes B-cell-activating factor (BAFF) signaling and supports the formation of germinal center and antibody production (Sabapathy et al., 1999; Huang et al., 2009). Overall, JNK acts as a central regulator, balancing T and B cell responses in order to optimize protective adaptive immunity during Mtb infection.

When Mtb PAMPs are recognized by TLRs, the TAK1–MKK4/7–JNK cascade is activated in macrophages. This results in the activation of transcription factors (c-Jun, p53, Beclin-1, ATF2, c-Myc, and SMAD), the production of pro-inflammatory cytokines (IL-1β, TNF-α, and IL-6) and apoptosis through mitochondrial and extrinsic pathways. Antigen presentation by dendritic cells subsequently triggers T-cell activation. JNK1 promotes Th1 differentiation by inhibiting Itch-mediated Th2 bias. Conversely, JNK1 enhances and JNK2 suppresses CD8^+^ T-cell proliferation by regulating IL-2Rα. These coordinated effects contribute to the immune clearance of Mtb.

## 4 The role of dysregulated JNK signaling in the pathogenesis of tuberculosis and the regulatory role of other MAPK pathways

During infection, Mtb enhances its intracellular survival by manipulating host metabolism and immune responses via the JNK signaling pathway. Under physiological conditions, JNK signaling pathway plays a crucial role in maintaining the balance between immune defense and metabolic homeostasis. However, Mtb infection disrupts this balance to facilitate bacterial persistence and latent infection. Dysregulation of JNK signaling, characterized by abnormal activation, excessive production of inflammatory mediators, impaired autophagy and induction of immune tolerance, ultimately compromises host immunity and hampers the clearance of intracellular pathogens, potentially leading to disease deterioration. In addition to JNK, other MAPK pathways, such as p38 and ERK, are involved in the host response to Mtb infection. Depending on the cell type and infection stage, their activation or inhibition can mediate either protective immune responses or pathological inflammation (see **Table 1**).

**Table 1:** Regulation of the p38/ERK MAPK pathway by Mtb and its immunological consequences.

Pathway	Regulation of pathway by mtb	Immunological consequence
P38 MAPK	Neutrophils from patients with active TB recognize Mtb through TLR2, activating the p38 MAPK pathway. This pathway induces apoptosis and contributes to anti-tuberculosis immune regulation (Alemán et al., 2004).	The activation of p38 promotes neutrophil apoptosis and plays a role in the host’s immune response to Mtb (Alemán et al., 2004).
P38 MAPK	An Mtb infection upregulates the expression of GM-CSF via the PI3-K/MEK1/p38 MAPK pathway, thereby promoting the recruitment, activation and formation of granulomas by macrophages (Cho et al., 2013).	It enhances the recruitment of macrophages and the formation of inflammatory granulomas (Cho et al., 2013).
P38 MAPK	The Mtb EsxB protein inhibits p38 autophosphorylation while activating METTL14. This blocks m6A modification and decreases ROS generation, thereby promoting pathogen survival (Ma et al., 2024).	Inhibiting p38 signaling weakens the host’s oxidative defense and facilitates Mtb survival (Ma et al., 2024).
ERK MAPK	Rv2140c mimics the function of mammalian RKIP, inhibits the phosphorylation of MEK1, ERK1/2, and IKKα/β, suppressing the activation of the ERK/NF-κB pathway. This reduces the production of proinflammatory cytokines (IL-1β, IL-6, and TNF-α), which favors the survival of Mtb in macrophages (Abo-Kadoum et al., 2021b).	Inhibiting ERK signaling reduces inflammatory cytokine production and promotes intracellular Mtb persistence (Abo-Kadoum et al., 2021b).
ERK MAPK	Blocking ERK (using the U0126 inhibitor/Tpl2-/-cells) inhibits IL-10 production and enhances IL-12p40 production in macrophages, but decreases IL-12p40 production in dendritic cells (Groft et al., 2019).	ERK signaling limits excessive inflammation in macrophages while restraining Th1 polarization in dendritic cells (Groft et al., 2019).
ERK MAPK	In TB patients, the MEK/ERK pathway regulates FoxP3 expression and Treg function. However, MEK inhibition by trametinib not only reduces Treg activation, but also reduces the expression of IFN-γ, TNF-α, and IL-2 expression in antigen-specific effector T cells. This suggests that the MEK/ERK pathway has a dual role in balancing immune activation and regulation (Lieske et al., 2015).	MEK/ERK signaling modulates both Treg activity and effector T-cell cytokine production, thereby maintaining immune homeostasis during Mtb infection (Lieske et al., 2015).

### 4.1 Metabolic reprogramming of macrophages

Alveolar macrophages are important immune cells that play a critical role in the host’s immune defense against Mtb. They kill intracellular Mtb by secreting reactive oxygen species (ROS) and reactive nitrogen species (RNS), as well as initiating phagosome-lysosome fusion, autophagy, and apoptosis (Ehrt and Schnappinger, 2009). During Mtb infection, these alveolar macrophages undergo metabolic reprogramming, changing their energy metabolism from oxidative phosphorylation (OXPHOS) to glycolysis and fatty acid metabolism. Glycolysis-related genes are significantly upregulated in this process (Shi et al., 2015; Ogger and Byrne, 2021). Glycolysis is a key metabolic pathway that provides energy and enhances the antibacterial activity of alveolar macrophages by promoting the production of inflammatory mediators such as IL-1β) (Gleeson et al., 2016; Kim et al., 2020). The JNK signaling pathway regulates both glycolysis and fatty acid oxidation. For example, JNK1-mediated phosphorylation of Bad (a Bcl-2 family protein) at Thr-201 maintains the activity of the key glycolytic enzyme phosphofructokinase-1 (PFK-1), thereby supporting normal glycolytic metabolism and cell survival (Deng et al., 2008). In addition, in the mouse Mtb infection model, upregulation of PKM2 (pyruvate kinase M2 isoform), a key rate-limiting enzyme in glycolysis, was detected in macrophages and mouse lung tissue, it is essential for LPS-induced DC activation (Shi et al., 2015). JNK-dependent acetylation of PKM2 at Lys433 promotes glycolysis and fatty acid synthesis, thereby driving dendritic cell metabolic reprogramming and activation (Zhu et al., 2021). Furthermore, JNK mediates lipopolysaccharide (LPS)-induced HIF-1α mRNA expression in monocytes, macrophages and hepatocytes, thereby linking inflammatory and metabolic responses (Frede et al., 2006).

Within the granulomatous microenvironment of Mtb infection, the bacterium obtains energy through the β-oxidation of fatty acids, the metabolism of ketone bodies and cholesterol, to enhance its ability to survival amid hypoxia and nutrient deprivation (Davis et al., 2024). In addition, Mtb can use iron as a nutrient source to support its survival and proliferation further (Boelaert et al., 2007; Olakanmi et al., 2007). Studies have shown that Mtb infection disrupts host lipid metabolism, increasing the levels of circulating free fatty acids and inducing ectopic lipid deposition in organs important for glucose homeostasis, such as the liver and skeletal muscle. This promotes insulin resistance (Bisht et al., 2023). The JNK signaling pathway is considered an important regulator; its sustained activation in adipose tissue, the liver, muscle, the pancreas, and the central nervous system promotes chronic inflammation and exacerbates metabolic dysfunction (Yung and Giacca, 2020). Severe insulin resistance can lead to diabetic ketoacidosis (DKA) (Dhillon and Gupta, 2025). Interestingly, clinical observations report a higher incidence of TB in patients with recurrent DKA, suggesting a bidirectional relationship between the two diseases. JNK activation may play a bridging role in the vicious cycle between DKA and TB. The chronic inflammatory environment caused by TB activates JNK signaling, leading to insulin resistance and excessive ketone body production, which significantly increases the risk of DKA (Hirosumi et al., 2002). Conversely, DKA weakens the patient’s immune function, exacerbating TB progression and creating a feedback loop between the two diseases (Al-Rifai et al., 2017).

In summary, the various ways in which the JNK signaling pathway regulates immune cell metabolism highlight its important role in TB. Under the metabolic stress of Mtb infection, abnormal JNK signaling can weaken the host’s antibacterial immune response and intensify the interaction between TB and metabolic disorders (such as DKA). This can promote disease progression. This discovery provides a new perspective for developing strategies to regulate metabolism and potential host-directed therapeutic approaches against Mtb infection.

### 4.2 Mtb weakens host immune responses by inhibiting the JNK signaling pathway

Many pathogenic bacteria have evolved complex strategies to avoid elimination by interfering with the host innate immune signaling pathways (Reddick and Alto, 2014). During infection, Mtb expresses a variety of virulence factors, such as Mce2E and PtpA, that weaken the host’s innate immune response by inhibiting JNK signaling. This promotes bacterial survival and proliferation within host cells.

The Mce2E operon contributes to Mtb pathogenity by directly inhibiting the JNK signaling cascade. Mce2E inhibits JNK activation by either blocking its interaction with upstream kinases or by sequestering JNK1 within the endoplasmic reticulum (ER), thereby inhibiting the host immune response to mycobacterial infection (Qiang et al., 2019). In addition, the Mtb tyrosine phosphatase PtpA is an important secreted effector that specifically inhibits JNK activation in a phosphatase activity-dependent manner. PtpA inhibits phagosome-lysosome fusion and blocks phagosome acidification by doing so, effectively attenuating the host cell autonomous immunity (Wang et al., 2015; Wawrocki and Druszczynska, 2017). Due to its vital function in the intracellular survival and virulence, the pharmacological inhibition or genetic disruption of PtpA has been demonstrated to restore the activity of the host JNK signaling pathway and enhance the host immune defenses against Mtb (Bach et al., 2006; Bach et al., 2008; Wong et al., 2011). Recent work by Wang J et al. showed that the host protein TRIM27 limits mycobacterial survival in macrophages by promoting JNK pathway activation and the apoptosis (Reddick and Alto, 2014). However, PtpA counteracts this defense mechanism by competitively binding to the RING domain of TRIM27, thereby antagonizing TRIM27-mediated JNK activation and apoptosis, thereby enhancing pathogen survival (Wang et al., 2016). Based on this, targeting the TRIM27-PtpA interaction interface may become a potential new strategy for the treatment of tuberculosis (Wang et al., 2016). Targeting the TRIM27–PtpA interaction interface thus represents a promising host-directed therapeutic approach for TB. Taken together, these findings emphasize that Mtb-mediated inhibition of JNK signaling is a key immune evasion strategy. Elucidating the molecular basis of this suppression and developing interventions that restore JNK pathway activity could lead to new approaches for treating TB.

### 4.3 Effect of the JNK signaling pathway on apoptosis during Mtb infection

Accumulating evidence suggests that the dysregulation of the JNK signaling pathway plays an important role in the pathogenesis of TB by regulating apoptosis. Macrophage apoptosis induced by Mtb infection primarily occurs through two classical mechanisms of apoptosis. The extrinsic pathway is triggered when Mtb activates tumor necrosis factor (TNF) receptor family members, initiating apoptotic signaling at the cell surface, and then activates the cascade of apoptotic caspase (Paik et al., 2019). The intrinsic apoptosis pathway involves the upregulation of the pro-apoptotic protein Bax, which promotes the release of cytochrome c from the mitochondria, and activates the caspase-9/3 cascade, ultimately leading to apoptosis (Datta et al., 2018). Following Mtb infection, macrophages can polarize into either M1 or M2 phenotypes in response to distinct cytokines environments, and this polarization largely determines their functional outcomes. M1 macrophages enhance inflammatory responses, promote apoptosis and facilitate Mtb clearance, whereas M2 macrophages reduce inflammation and apoptosis, maintain immune tolerance, and inhibit excessive immune activation (Lisnock et al., 2000; Lim et al., 2016). Interestingly, macrophages infected with the avirulent Mtb strain H37Ra predominantly exhibit an M1 phenotype, whereas macrophages infected with the virulent strain H37Rv drives polarization towards an M2 phenotype (Lam et al., 2017). This difference reflects the distinct virulence capacities of the two strains: H37Ra more readily induces pro-inflammatory and pro-apoptotic responses that aid bacterial clearance, while H37Rv promotes an anti-inflammatory, apoptosis-resistant state that favors immune evasion. Therefore, the virulence of Mtb critically determines whether macrophages undergo apoptosis to control infection or tolerance to permit bacterial persistence.

During Mtb infection, cytokine dynamics further modulate macrophage apoptosis. A meta-analysis showed that IL-4 levels were significantly increased in the serum and bronchoalveolar lavage fluid of TB patients (He et al., 2023). IL-4 inhibits Th1 immune responses and reduces the ability of macrophages to clear Mtb (Jeevan et al., 2011; Pooran et al., 2019). IL-4 promotes M2 polarization by activating the JNK signaling pathway, thereby inhibiting apoptosis and weakening host defenses (Hao et al., 2017; Pooran et al., 2019).

In addition to cytokine regulation, apoptosis is modulated by JNK-interacting proteins. The host protein phosphatase-magnesium/manganese-dependent 1A (PPM1A) is markedly upregulated following Mtb infection. Excessive production of PPM1A inhibits the apoptotic response of macrophages by dephosphorylating and inactivating the JNK signaling pathway, ultimately impairing the clearance of Mtb (Schaaf et al., 2017). PPM1A inhibits the activation of the JNK downstream transcription factor ATF2 by dephosphorylating JNK (Rajabalee et al., 2023). Overexpression of ATF2 significantly enhance the antibacterial effect of M1 macrophages against Mtb infection (Rajabalee et al., 2023). Therefore, JNK, as a key regulator of PPM1A and ATF2 activity, plays an important role in the immune response to Mtb infection by affecting macrophage apoptosis.

However, excessive apoptosis induced by Mtb infection may also allow Mtb to evade host immune clearance. Studies have shown that the 19 kDa lipoprotein (P19) secreted by Mtb can induce macrophage apoptosis, thereby contributing to Mtb immune escape (Li et al., 2014). As a potential anti-TB drug, low-dose curcumin can protect macrophages from P19-induced apoptosis by inhibiting JNK activation and reducing cytokine overproduction (Li et al., 2014). Furthermore, treatment with a JNK-specific inhibitor enhances the protective effect of curcumin on macrophage viability, suggesting that controlled inhibition of JNK activity can prevent excessive apoptosis and limit Mtb dissemination (Li et al., 2014).

In summary, the JNK signaling pathway plays a dual role in Mtb infection. It promotes host’s antibacterial defenses by regulating the apoptosis process of macrophages; however, it can also be used by Mtb to evade the host immune clearance. Understanding this bidirectional regulatory mechanism establishes JNK as a key target in the study of Mtb immune evasion and the development of host-directed TB therapies.

### 4.4 Effect of JNK pathway dysregulation on autophagy regulation in Mtb infection

Autophagy is an important defense mechanism of the host that eliminates intracellular pathogens, such as Mtb, thereby limiting bacterial proliferation and tissue injury (Deretic et al., 2009; Jo, 2013; Liu et al., 2017; Chai et al., 2020). Studies show that macrophages from patients with active tuberculosis (ATB) exhibit enhanced autophagic activity following Mtb infection (Li et al., 2019; Ke et al., 2020; Shi et al., 2020). Under physiological conditions, autophagy contributes to intracellular homeostasis and pathogen clearance, thereby preventing excessive cell death and maintaining host immune defense. However, dysregulated autophagy enables Mtb to evade immune clearance and persist within host cells (Jurkuvenaite et al., 2015). One of the key Mtb virulence factors involved in this process is the enhanced intracellular survival (eis) gene. Eis protein negatively regulates autophagy, inflammation, and cell death in macrophages by inhibiting the production of reactive oxygen species (ROS) through the JNK-dependent pathway, thereby weakening host immune function and promoting Mtb survival (Shin et al., 2010). However, infection with the Mtb H37Rv eis-deficient mutant (Mtb-Δ*eis*) can induce excessive autophagy and inflammation, and significantly increased ROS levels, leading to caspase-independent cell death (Shin et al., 2010). This results in organelle damage and impaired bacterial clearance (Shin et al., 2010). Further, mechanistically, JNK activation is a key step in Mtb-Δ*eis*-induced ROS generation and cell death; conversely, inhibition of the JNK–ROS axis significantly reduces this cytotoxic effect (Shin et al., 2010). These findings suggest that balanced JNK activity is essential for maintaining effective, non-lethal autophagy during infection.

Although there is limited direct evidence for JNK-mediated regulation of autophagy during Mtb infection, studies in other cellular systems provide valuable insights into the underlying mechanisms. For example, in cancer cells, JNK activation induces Beclin-1 expression and promotes the formation of autophagosome, resulting in autophagy-associated cell death (Li et al., 2009). Similarly, ROS-triggered JNK activation in cardiomyoblasts enhanced autophagy and cell death (Younce and Kolattukudy, 2010). In addition, JNK has been shown to upregulate Atg7, thereby inducing non-canonical autophagy and enhancing autophagic flux in tumor models (Wong et al., 2010). Notably, Atg7 is a key protein in the formation of autophagosomes. Activation of JNK is a necessary condition for Atg7 expression; conversely, loss of Atg7 prevents autophagosome formation and limits Mtb replication in macrophages (Wong et al., 2010; Aylan et al., 2023). While a direct mechanistic link between JNK and Atg7 has yet to be demonstrated in TB, evidence from Mtb-infected macrophages clearly shows that Atg7 is essential for limiting bacterial replication. Collectively, these findings suggest that a JNK–Atg7 regulatory axis may represent an important, yet as yet unexplored, mechanism underlying host autophagic defense against Mtb infection.

Beclin-1, another key regulator of autophagy, is elevated in alveolar macrophages from TB patients, and higher Beclin-1 levels correlates with enhanced bacterial clearance (Pellegrini et al., 2021). This process may be related to JNK activation, whereby JNK phosphorylates Bcl-2, resulting in the dissociation of the Bcl-2/Beclin-1 complex and subsequent release of Beclin-1 to initiate autophagy (Maundrell et al., 1997; Wei et al., 2008). These findings suggest that JNK may promote autophagy by disrupting the Bcl-2–Beclin-1 interaction, thereby contributing to the host’s defense against Mtb. However, Mtb has evolved strategies to subvert this defense mechanism (Maundrell et al., 1997; Wei et al., 2008). The Mtb Eis protein, an ϵ-aminoacetyltransferase, acetylates a JNK-specific phosphatase, thereby enhancing its activity. This results in the dephosphorylation and subsequent inactivation of JNK. This suppresses Beclin-1-mediated autophagy, helping Mtb to evade immune clearance and maintain a persistent infection (Shin et al., 2010; Ganaie et al., 2011; Kim et al., 2012). Therefore, while JNK-mediated activation of Beclin-1 enhances the host’s antibacterial response, excessive or dysregulated JNK–Beclin-1 signaling may, conversely, impair immune homeostasis and create an environment favorable to long-term Mtb survival.

In summary, although direct evidence for JNK-regulated autophagy during Mtb infection remains limited, existing data indicate that JNK functions as a pivotal node linking oxidative stress, Beclin-1 activation and Atg7-dependent autophagic responses. Maintaining appropriate JNK activity appears essential for efficient antibacterial autophagy whilst preventing excessive cell death. Further mechanistic studies are needed to elucidate how Mtb manipulates JNK-mediated signaling to fine-tune autophagy for immune evasion.

### 4.5 Effect of JNK pathway dysregulation on ferroptosis in Mtb infection

Ferroptosis is an iron-dependent form of regulated cell death driven by lipid peroxidation. Increasing evidence suggests that ferroptosis may create a microenvironment favorable to Mtb survival and proliferation in host cells by weakening the host immune defense. Autophagy can promote ferroptosis by selectively degrading ferritin (ferritinophagy) (Hou et al., 2016), and ferritin deficiency is closely related to severe pulmonary pathology in Mtb-infected mice (Reddy et al., 2018). In macrophages, excessive intracellular iron accumulation is accompanied by the release of ROS via the Fenton reaction, driving lipid peroxidation and ferroptosis (Yang et al., 2022). While there is a lack of direct evidence of JNK-mediated ferroptosis in TB, studies from other disease models have provided important clues. In various cancer and oxidative stress models, activation of the RAS–JNK/p38 signaling cascade upregulates transferrin receptor 1 (TfR1), enhancing iron uptake and making cells more susceptible to ferroptosis under oxidative conditions (Ye et al., 2019). In acetaminophen-induced liver injury, JNK directly regulate the stability of the antioxidant transcription factor Nrf2 via phosphorylation. Specifically, phosphorylated JNK (p-JNK) targets Ser-335 within the DSGIS motif of Nrf2 (residues 334–338), promoting its degradation in a Keap1-independent manner and resulting in a negative correlation between intracellular Nrf2 levels and p-JNK activity (Chen et al., 2020). The Nrf2 pathway is known to exert a potent anti-ferroptotic effect, particularly in models of acute lung injury. Activation of Nrf2 upregulates key antioxidant genes such as SLC7A11 and HO-1, thereby maintaining glutathione homeostasis and reducing lipid peroxidation (Dong et al., 2020). Consistent with these findings, elevated systemic iron levels are associated with an increased risk of active pulmonary TB in patients and an increased bacterial load in Mtb-infected mice. This highlights the strong correlation between iron-dependent ferroptosis and TB pathogenesis (Schaible et al., 2002; Boelaert et al., 2007). Taken together, these findings suggest that JNK may regulate ferroptosis via two main mechanisms: (1) promoting intracellular iron accumulation via TfR1 upregulation and (2) regulating antioxidant defenses(by suppressing Nrf2 activity. In support of this, the specific JNK inhibitor SP600125 effectively block ferroptosis induced by recombinant LCN2 (rLCN2) in retinal photoreceptors, indicating that JNK activity is required for the ferroptosis process (Tang et al., 2025).

In summary, JNK may act as a key regulator of ferroptosis during Mtb infection. Dysregulation of JNK activation may exacerbate the ferroptotic damage in macrophages, thereby promoting Mtb immune escape and persistence. However, further mechanistic and *in vivo* studies are needed to verify the precise role of JNK-mediated ferroptosis in TB pathogenesis.

### 4.6 Inhibitory effect of JNK pathway dysregulation on the secretion of inflammatory cytokine during Mtb infection

During Mtb infection, dysregulation of the JNK signaling pathway has a significant impact on the host immune response, particularly with regard to the secretion of pro-inflammatory cytokines. Under physiological conditions, moderate JNK signaling activation can induce macrophages to release TNF-α, IL-6, and IL-1β, thereby contributing to pathogen clearance. However, Mtb can inhibit JNK activity through specific virulence proteins, thereby interfering with cytokine release, weakening immune surveillance and enabling the bacterium to persist in a latent or chronic state. Beyond JNK, other MAPK branches also contribute to Mtb-induced inflammation. For example, the ESAT-6–like protein EsxL has been shown to interact with TLR2 and induces the phosphorylation of ERK and p38. Inhibition of ERK or p38 significantly reduces recombinant EsxL-induced TNF-α production in macrophages, and silencing TLR2 further diminishes TNF-α release (Pattanaik et al., 2021). These findings highlight that Mtb manipulates an interconnected signaling network including JNK, ERK, and p38 pathways rather than a single linear cascade to fine-tune host inflammatory responses.

Several secreted or surface-exposed proteins secreted by among Mtb’s virulence factors play a pivotal role in suppressing JNK signaling. The LprG protein encoded by the *Rv1411c* gene, a surface glycolipoprotein of Mtb, not only contributes to bacterial virulence but also directly inhibits JNK activation (Abekura et al., 2022). Mechanistic studies have shown that LprG significantly inhibits inflammatory responses in macrophage by interfering with the TLR2-dependent JNK pathway. This suppression is characterized by reduced expression of inducible nitric oxide synthase (iNOS), the enzyme responsible for nitric oxide (NO) production, together with diminished release of TNF-α, IL-6 and other pro-inflammatory cytokines, thereby weakening the host immune defenses against Mtb and facilitating Mtb persistence during latent infection (Abekura et al., 2022). Similarly, the Rv2387 protein has been identified as another key effector that attenuates inflammatory responses in macrophages via TLR2-dependent inhibition of the JNK pathway, thereby allowing mycobacterial immune evasion (Li et al., 2022). Additionally, the Mtb cell wall protein Rv0309 exerts inhibitory effects on the production of pro-inflammatory factors through modulation of JNK signaling. Peng Y et al. showed that Rv0309-mediated inhibition of the JNK pathway exacerbates pulmonary lesions and increases bacterial burden in infected hosts (Peng et al., 2022).

**Figure 4:**
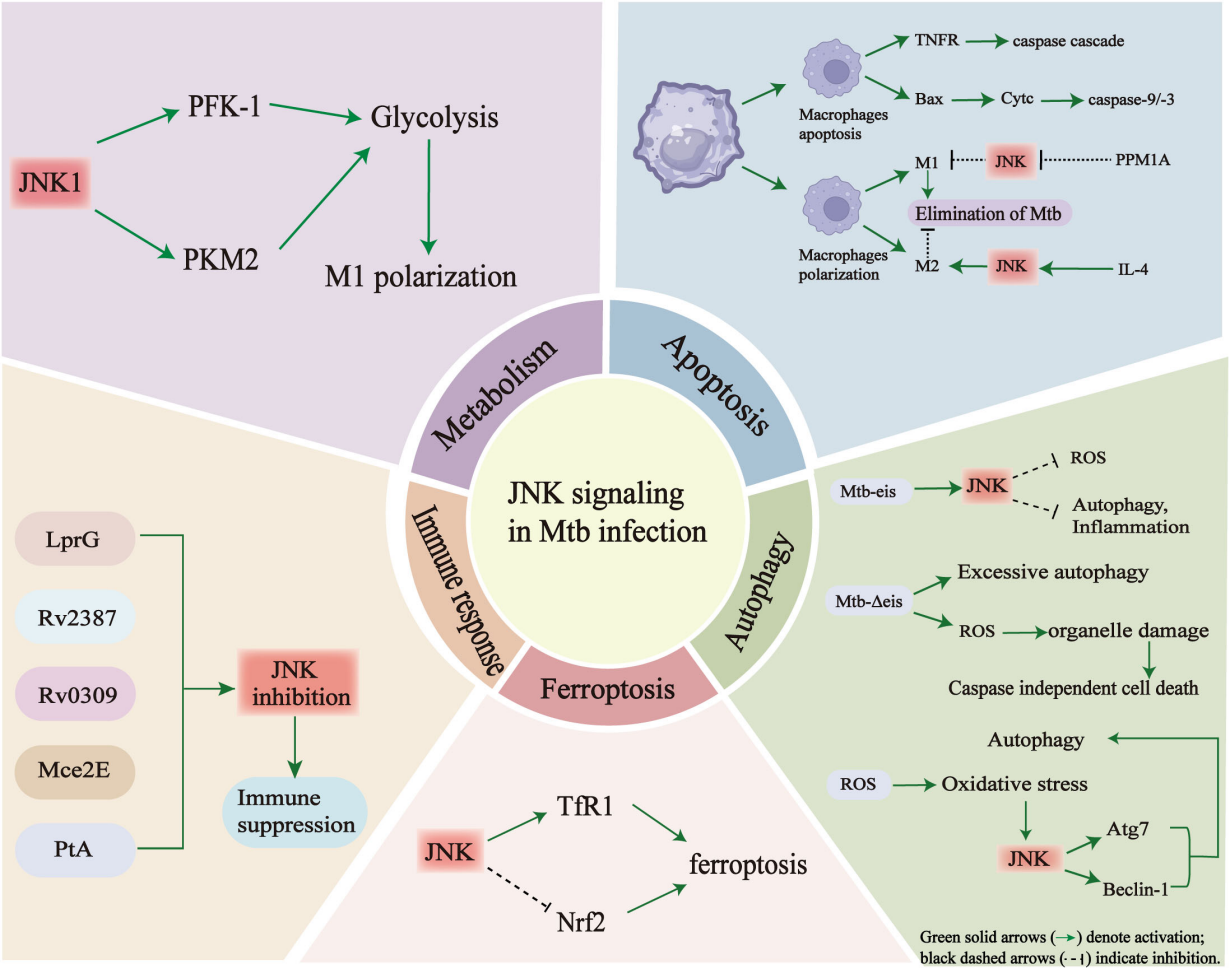
The multifaceted roles of JNK signaling in Mtb infection.

In summary, Mtb inhibits host inflammatory responses by secreting multiple effector proteins that inhibit JNK signaling pathway activation. This significantly reduces the release of key pro-inflammatory cytokines and iNOS expression, thereby weakening antibacterial activity in macrophages and facilitating bacterial survival. The ability of Mtb to manipulate JNK-mediated cytokine regulation represents a crucial mechanism of immune evasion and offers potential therapeutic intervention targets. As shown in **Figure 4**, the JNK signaling pathway plays a key role in coordinating in cell metabolism, immune response, apoptosis, autophagy, ferroptosis, and cytokine secretion during Mtb infection.

JNK signaling acts as a central regulator, linking metabolism, apoptosis, autophagy, ferroptosis, and immune responses through multiple molecular interactions during Mtb infection. (Top left) In alveolar macrophages, JNK1 activation shifts energy metabolism from oxidative phosphorylation (OXPHOS) towards glycolysis by promoting PFK-1 and PKM2 activity. This leads to increased glycolytic flux and inflammatory polarization. However, persistent JNK activation may also contribute to insulin resistance and metabolic dysfunction, thereby weakening host immunity. (Top right) In the context of apoptosis, JNK mediates pro-apoptotic signaling through TNFα, Bax and cytochrome c release, activating caspase-9/3 cascades. JNK also influences macrophage polarization: activation favors M1 differentiation and Mtb elimination, while inhibition (e.g. via PPM1A) promotes M2 polarization and bacterial persistence. (Lower right) During autophagy, Mtb-secreted effectors such as Eis can trigger JNK-dependent ROS accumulation, leading to excessive autophagy and inflammation. Overactivation of this pathway may result in organelle damage and caspase-independent cell death. JNK can also regulate key autophagy mediators such as Atg7 and Beclin-1. (Lower center) JNK contributes to ferroptosis by increasing TfR1 expression and suppressing Nrf2 activity. This enhances iron-dependent lipid peroxidation and oxidative stress. (Lower left) Multiple Mtb proteins, including LprG, Rv2387, Rv0309, Mce2E and PtA, interact with or modulate JNK signaling to suppress host immune activation, thereby facilitating immune evasion. Taken together, these findings emphasize the pivotal role of JNK in integrating metabolic reprogramming, cell death and immune responses during TB pathogenesis.

### 4.7 Dysregulation of the JNK signaling pathway exacerbates the inflammatory response to Mtb infection

Pulmonary TB is characterized by chronic inflammation. While acute inflammation initially aids in pathogen containment, an unresolved Mtb infection drives sustained chronic inflammation, leading to progressive lung tissue damage marked by necrosis, fibrosis and functional impairment. These pathological outcomes result from prolonged immune activation and dysregulated inflammatory cascades (Ravimohan et al., 2018).

Following inhalation, Mtb is phagocytosed by alveolar macrophages and subsequently translocated to the lung interstitium. Although early infection is partially controlled by innate immunity, Mtb uses multiple immune evasion strategies, including the suppression of macrophage antimicrobial activity, - to establish long-term intracellular survival (Killick et al., 2013; Arnett et al., 2023). Persistent Mtb infection drives the sustained production of pro-inflammatory cytokines (e.g., TNF and IL-6) and chemokines, recruiting additional immune cells to form granulomas. While granulomas simultaneously provide protection and pathology: they limits Mtb replication and dissemination; however, prolonged granulomatous inflammation promotes tissue remodeling, necrosis, and hypoxic microenvironments that facilitate bacterial reactivation (Tiwari and Martineau, 2023).

Cytokine dysregulation plays a key role in these pathological changes. High concentrations of TNF-α strongly correlate with inflammatory severity and bacterial burden in animal models (Bekker et al., 2000). This suggests that the role of TNF-α in TB is both protective and can also induce excessive inflammatory responses, depending on its local concentration (Bekker et al., 2000). In an Mtb-infected rabbit model, treatment with TNF antagonists (e.g., etanercept) exacerbated pulmonary pathology and impaired bacillary control, underscoring that TNF-α signaling is indispensable for collagen/fibrin deposition, granuloma organization, and pathogen containment (Tsenova et al., 2014). Clinical evidence further shows that excessive production of inflammatory mediators exacerbates TB pathology. Mechanistically, TNF-α binds to TNFR1 or TNFR2, activates the JNK and NF-κB signaling pathways, leading to the transcription of multiple pro-inflammatory genes (Domingo-Gonzalez et al., 2016). JNK and NF-κB share common upstream activators and act synergistically in the inflammatory response. JNK promotes NF-κB activation by degrading IκB-α, thereby accelerating the transcription of inflammatory genes and the infiltration of immune cells (Deretic, 2014; Poladian et al., 2023). However, when JNK activation becomes excessive, this positive feedback loop can escalate into harmful immunopathology. Chronic inflammation also alters the metabolism of the extracellular matrix (ECM) in the lung. Excessive collagen deposition induces fibrosis, stiffens lung tissue and impairs pulmonary function (Kim and Putyatina, 2023). Evidence from other respiratory infections supports the pathological role of the JNK pathway. For example, during influenza A virus infection, JNK enhances the expression of the inflammation amplifier TREM1, exacerbating the inflammatory storm (Chen et al., 2025). Similarly, overactivation of JNK may drive excessive inflammation and lung damage in TB, thereby weakening host resilience (Chen et al., 2025). Mihaltan previously suggested that TNF-α blockade might mitigate TB inflammation (Mayordomo et al., 2002; Mihăltan, 2007). However, more recent evidence from animal studies indicates that such treatment exacerbates pathology and reduces bacillary control. This highlights the essential role of TNF-α/JNK signaling in granuloma maintenance and host defense (Mayordomo et al., 2002; Mihăltan, 2007; Tsenova et al., 2014). Interestingly, Mtb exploits this pathway in the early phase of infection through virulence factors such as LprG, which suppress JNK signaling to promote immune evasion (Abekura et al., 2022). These findings emphasize the need for precise modulation of JNK inhibition as a therapeutic strategy. The aim of JNK-targeted host-directed therapy is not to mimic Mtb-induced immune suppression, but rather to reduce excessive inflammation at later stages of the disease when uncontrolled JNK activation causes tissue damage. Therefore, treatment efficacy depends on the timing and dosage of selective targeting of pathological rather than protective JNK activity.

In summary, the JNK signaling pathway can become dysregulated in TB in two opposing ways, both of which are detrimental to the host. On the one hand, overactivation of JNK can amplify the production of pro-inflammatory cytokines (e.g., TNF-α and IL-6), exacerbates the recruitment of immune cell, and contributes to tissue damage, fibrosis, and functional impairment. On the other hand, diminished JNK activity, mediated by Mtb virulence factors such as LprG, reduces the antimicrobial effector functions of macrophages, weakening the host’s resistance, and promoting bacterial persistence. Therefore, future therapeutic strategies should focus on the precise and stage-specific regulation of JNK activity rather than simple inhibition or activation, to restore immune balance and minimize TB-associated tissue damage. This approach could help to control inflammation while preserving essential antimicrobial functions.

## 5 Potential therapeutic strategies

Although antibiotics remain the mainstay of TB treatment, the current treatment regimen faces many challenges, such as poor patient compliance caused by long-term multidrug combination therapy, adverse drug reactions, and the increase in drug resistance (Mi et al., 2021). In recent years, host-directed therapy (HDT), which enhances the host immune response, improve antibacterial efficacy and accelerate the resolution of inflammation through small-molecule modulators, has emerged as a promising complementary approach. HDT helps to reduce the occurrence of drug resistance and treatment-related complications, and has therefore become a research hotspot in the field of TB therapeutics (Mi et al., 2021). The JNK signaling pathway plays a key role in regulating cellular stress, inflammatory responses, apoptosis, and autophagy (Shen and Liu, 2006; Weston and Davis, 2007). There is increasing evidence that aberrant JNK activation is closely associated with the onset and progression of TB and several inflammatory diseases (e.g., rheumatoid arthritis, inflammatory bowel disease and asthma). In pre-treatment TB patients, overactivation of JNK drives aberrant and excessive inflammatory responses, leading to immune dysregulation, severe tissue damage and fibrosis (Deretic, 2014; Domingo-Gonzalez et al., 2016; Chen et al., 2018; Kim and Putyatina, 2023; Poladian et al., 2023). Therefore, the pharmacological inhibition of JNK is a rational strategy for attenuating excessive inflammation and limiting tissue damage during Mtb infection (Cho et al., 2012; Lee et al., 2019).

A variety of highly potent and selective JNK inhibitors have been developed, mainly including two categories: (1) ATP-competitive inhibitors, such as SP600125 (Wu et al., 2015; Shen et al., 2017), bentamapimod (Palmer et al., 2016), CC-930 (Plantevin Krenitsky et al., 2012; Graczyk, 2013), and CC-359 (Plantevin Krenitsky et al., 2012; Graczyk, 2013), and (2) JNK-interacting protein (JIP)-binding inhibitors, such as BI-78D3 (Stebbins et al., 2008) and SU3327 (Jang and Javadov, 2014). These inhibitors have demonstrated robust anti-inflammatory and anti-fibrotic effects in preclinical models of lung injury, asthma, and pulmonary fibrosis, with several advancing to clinical trials. However, the pan-JNK inhibitor CC-930 (tanzisertib) was terminated in Phase II trials due to hepatotoxicity, underscoring the safety concerns of broad JNK blockade (van der Velden et al., 2016). Notably, the JNK inhibitor SP600125 inhibits intracellular Mtb replication at low concentrations (1 μM) and exhibits synergistic effects when combined with the translation inhibitor RocA (Yabaji et al., 2023). This combination reprograms transcriptional state of macrophages and significantly enhances their ability to control the highly virulent Mtb (Yabaji et al., 2023). Furthermore, Liang et al. reported that treatment with the traditional Chinese medicine compound NiuBei XiaoHe extract downregulated JNK signaling and alleviated excessive pulmonary inflammation in a murine TB model, providing preliminary evidence of the therapeutic potential of JNK modulation in TB (Ling et al., 2016). Although the delivery of JNK inhibitors to macrophages or the lungs has not yet been reported in TB, results from other disease models support its feasibility. For example, D-JNKi-1nanoparticles achieved localized delivery in cochlear cells (Kayyali et al., 2018), and SP600125 nanoparticles have successfully crossed the blood–brain barrier to enhance radiosensitivity in brain tumors (Li et al., 2023). Such delivery strategies could be adapted for TB to achieve localized immune regulation and improved antimicrobial efficacy. Despite their therapeutic potential, there are several challenges to the clinical translation of JNK inhibitors. The lack of isoform selectivity increases the risk of off-target effects; and poor oral bioavailability and the potential for the development of resistance further constrain their application (Feng et al., 2024). Future efforts should therefore focus on developing safer, isoform-selective JNK inhibitors with optimized pharmacokinetic properties (Feng et al., 2024). Furthermore, artificial intelligence (AI) technologies have transformed drug repurposing and antibacterial discovery. A prime example is Halicin, which was initially developed as a JNK inhibitor, but was later rediscovered through deep learning as an effective, broad-spectrum antibiotic against multidrug-resistant pathogens (El Belghiti et al., 2025). This demonstrates the potential of AI-assisted screening to reposition JNK inhibitor for novel anti-TB applications.

In addition to JNK, the p38 MAPK pathway also plays a crucial role in Mtb infection. Moderate activation of p38 induces protective immunity and promotes pathogen clearance, whereas excessive activation promotes inflammation. The anti-TB drug SQ109 has been shown to activate both the p38 and JNK pathways, thereby enhancing M1 macrophage polarization, apoptosis, and the production of cytokines such as IL-6, IL-12, and IFN-γ (Singh et al., 2022). Conversely, the p38 inhibitor dolamamod reduces lung acute inflammation and granuloma formation during acute infection, but suppresses key cytokines during chronic infection, potentially weakening antimicrobial immunity (Hölscher et al., 2020).

In line with the WHO “End TB Strategy,” integrating JNK/MAPK-targeted agents with AI-assisted drug repurposing and precision delivery technologies may open new avenues for host-directed TB therapies. Ultimately, fine-tuning MAPK activity, rather than broad inhibition, will be essential to balance protective immunity and prevent immunopathology. Continued development of selective JNK inhibitors, targeted delivery technologies, and AI-driven drug repurposing may provide promising avenues for host-directed therapies against TB.

## 6 Existing problems and prospects

Although significant progress has been made in elucidating the role of the JNK signaling pathway in the pathogenesis of TB and exploring its therapeutic potential, there are still many problems and challenges:

(1) The incomplete understanding of JNK signaling mechanisms in TB. The precise mechanism by which the JNK signaling pathway regulates host-pathogen interactions during Mtb infection are not fully elucidated. The JNK family has multiple isoforms (e.g., JNK1, JNK2, and JNK3), each of which involves in complex cellular processes such as inflammation, apoptosis, and autophagy, and interacts intricately with other key signaling pathways (e.g., NF-κB, MAPK, PI3K/Akt, etc). Furthermore, the cell-type-specific functions of JNK signaling in macrophages, dendritic cells, epithelial cells, and lymphocytes are not well understood. As these cell populations exhibit distinct JNK activation dynamics and immune outcomes, understanding their context-dependent regulatory mechanisms is essential for developing safe and effective host-directed therapies.

(2) Limited evidence on therapeutic applications. To date, only a few studies have investigated the use of JNK inhibitors in TB treatment. Their pharmacological efficacy, selectivity and safety profiles still need to be further verified through rigorous preclinical and clinical studies.

(3) Potential risk of immune imbalance. Given the dual roles of JNK signaling in host defense and inflammatory injury, excessive inhibition may weaken protective immunity and increase the risk of infection. Therefore, how to maintain immune homeostasis—avoiding both excessive immune activation and immunosuppression—remains a challenging problem that needs to be addressed.

The following aspects deserve further research and exploration in the future: (1) The mechanistic dissection of JNK signaling dysregulation: Further study should elucidate the precise molecular mechanism of JNK pathway dysregulation during Mtb infection, paying particular attention to the distinct roles and functional differences of JNK isoforms in immune modulation. Investigating how JNK interacts with other signaling pathways to orchestrate immune and inflammatory responses will facilitate the identification of more specific therapeutic targets. (2) The integration of multi-omics technologies: Using multi-omics technologies such as single-cell RNA sequencing and spatial transcriptomics, will help to delineate the dynamic regulatory network of the JNK signaling pathway at different pathological stages of TB. These analyses could reveal cell-type-specific effects, clarify temporal activation patterns, and strike a balance between the anti-inflammatory effect and the risk of immunosuppression. (3) Crosstalk with metabolism, cell death and tissue repair: Future studies should explore how JNK signaling integrates immune responses with metabolic reprogramming, ferroptosis and tissue repair processes. Elucidating these interactions could lead to new ways of preventing TB progression, post-infectious complications, and excessive tissue damage. (4) Rational drug design targeting JNK isoforms: Combining artificial intelligence with structure-based drug design technology could accelerate the design, screening and optimization of novel small-molecule inhibitors that selectively target specific JNK isoforms (JNK1, JNK2, and JNK3), thereby reducing off-target effects and improving treatment efficacy. (5) Combination therapy strategies: Exploring the combination of JNK inhibitors with existing anti-TB drugs could enhance bactericidal activity and modulate host immune responses in a synergistic manner. It is worth noting that rifamycins, especially rifampicin, are potent CYP3A inducers and their CYP3A4 induction is partly dependent on the JNK-PXR-CYP3A4 axis, concomitant JNK inhibition can perturb this induction, making exposure and clearance of co-administered CYP3A4 substrates less predictable and risking unstable efficacy, delayed culture conversion, and interpretative bias (Taneja et al., 2018). Further preclinical and clinical research is needed to evaluate the potential advantages of this approach in treating drug-resistant TB. (6) Clinical validation and personalized therapy: Well-designed clinical trials targeting specific JNK pathway regulators are essential to verify their safety, efficacy, and optimal therapeutic window in treating TB patients. Furthermore, developing personalized therapeutic regimens based on molecular and immunopathological profiling, particularly with regard to differences in JNK pathway dysregulation among patients, could enhance treatment precision and effectiveness. (7) JNK signaling in immune tolerance and chronic inflammation: Understanding the role of JNK in maintaining immune tolerance and mediating chronic inflammatory responses during latent or persistent TB infection could provide valuable insights into preventing disease reactivation and long-term tissue damage.

In summary, while substantial challenges remain, continued investigation into the role of JNK signaling pathway in TB pathogenesis holds great promise. A deeper understanding of its cell-type-specific functions, interaction with metabolic and cell death pathways, and contribution to immune tolerance and chronic inflammation will be essential. Integrating multi-omics technologies, AI-assisted drug discovery and personalized, host-directed therapeutic approaches may ultimately establish the JNK pathway as a pivotal target, paving the way for innovative, effective anti-TB interventions.

## 7 Conclusion

Following infection with Mtb, the activation level of the JNK pathway determines whether host responses are protective or pathological. Moderate JNK activation promotes antimicrobial defense through autophagy and cytokine regulation, whereas sustained overactivation drives immune exhaustion, tissue injury, and fibrosis. Future research should emphasize isoform-selective modulation of JNK activity and its crosstalk with other MAPK members to achieve context-dependent immune balance. Understanding these dynamic regulatory mechanisms will aid in the rational design of host-directed therapies and accelerate the clinical translation of JNK-targeted interventions. We position JNK blockade as a short, late-stage, biomarker-guided host-directed add-on—aimed at tempering excessive inflammation and preventing tissue injury—rather than as a substitute bactericidal therapy. Any combination therapy regimens involving JNK inhibitors should only be considered during the later stages of anti-TB treatment, within a narrow treatment window, and should be accompanied by stringent monitoring of pathway inhibition, immune dynamics and drug concentrations.
